# Quantifying avian inertial properties using calibrated computed tomography

**DOI:** 10.1242/jeb.242280

**Published:** 2022-01-04

**Authors:** Nicholas E. Durston, Yusuf Mahadik, Shane P. Windsor

**Affiliations:** Department of Aerospace Engineering, University of Bristol, Bristol BS8 1TR, UK

**Keywords:** 3D dynamics modelling, Centre of mass, Inertia tensor, Bird flight

## Abstract

Estimating centre of mass and mass moments of inertia is an important aspect of many studies in biomechanics. Characterising these parameters accurately in three dimensions is challenging with traditional methods requiring dissection or suspension of cadavers. Here, we present a method to quantify the three-dimensional centre of mass and inertia tensor of birds of prey using calibrated computed tomography (CT) scans. The technique was validated using several independent methods, providing body segment mass estimates within approximately 1% of physical dissection measurements and moment of inertia measurements with a 0.993 *R*^2^ correlation with conventional trifilar pendulum measurements. Calibrated CT offers a relatively straightforward, non-destructive approach that yields highly detailed mass distribution data that can be used for three-dimensional dynamics modelling in biomechanics. Although demonstrated here with birds, this approach should work equally well with any animal or appendage capable of being CT scanned.

## Abstract (precis)

**Summary:** Demonstration of the use of calibrated computed tomography to accurately quantify the three-dimensional centre of mass and moments of inertia of birds of prey.

## INTRODUCTION

Estimates of centre of mass (CoM) and mass moment of inertia (MoI) are necessary features of many studies in animal biomechanics and locomotion. The CoM informs questions of stability and balance while MoI relates angular accelerations and moments via Newton's second law. In terrestrial locomotion, for example, the CoM is required for studies of stability at rest and in motion, while limb MoI is utilised for quantifying the energetics of the limb swing phase (Biewener and Patek, 2018). In flying animals, CoM and MoI are crucial parameters for modelling stability and flight dynamics (Durston et al., 2019). Wing MoI plays a role in understanding the energetics of flapping flight (Berg and Rayner, 1995), and is used by bats for controlling low-speed head-over-heels manoeuvres (Bergou et al., 2015). Similar examples may be found for swimming, climbing, suspension and jumping (Biewener and Patek, 2018).

Methods for CoM estimation in animal cadavers typically fall into four categories: balance, suspension, scales and digital modelling (Macaulay et al., 2017). The first of these involves use of a balance board or straight edge to find segment or whole-organism CoM. With the suspension approach, the cadaver is hung from multiple orientations such that the intersection of superimposed vertical reference lines provides the position of the CoM (Kilbourne et al., 2016; Ros et al., 2015; Thomas and Taylor, 2001). An example of the scales approach involves laying the cadaver on a beam supported by two mass balances and solving the corresponding moment equation to obtain CoM (Kilbourne, 2013; Macaulay et al., 2017). The digital modelling approach utilises image contrast from volume scans such as computed tomography (CT), magnetic resonance imaging or photogrammetric reconstruction to segment the scan into regions of uniform density based on tissue type (i.e. air, muscle, bone) (Allen et al., 2009; Brassey and Sellers, 2014; Hutchinson et al., 2007; Peyer et al., 2015). A study into the accuracy of the last three approaches concluded that suspension methods yield lower accuracy and repeatability compared with scales and digital modelling (Macaulay et al., 2017).

Methods for MoI estimation in animal cadavers also typically fall into four categories: shapes, pendulum, strips and digital modelling. The shapes method models the anatomy as a series of shapes (i.e. either primitive shapes or a scanned cadaver outline), each of uniform density, whose combined MoI can be calculated analytically (Kilbourne et al., 2016; Meriam and Kraige, 2012; Tucker, 1992). Pendulum methods relate the time period of oscillation of the cadaver with MoI via a dynamic model (Kilbourne, 2013; Ros et al., 2015). The strips method uses the recorded masses and positions of dissected cadaver segments to calculate MoI (Kirkpatrick, 1990; Thollesson and Norberg, 1991; Berg and Rayner, 1995). The digital modelling approach described above can also yield MoI estimates, although the magnitude of the error due to local variation in tissue density is unclear (Macaulay et al., 2017). The error associated with the primitive shapes and pendulum approaches is also unclear because studies often do not provide validation data. The accuracy of the strips method can be improved by increasing the number of strips, but this can also lead to increased damage caused by the dissection procedure.

Here, we describe a method for the generation of high spatial resolution three-dimensional (3D) mass distribution/inertial models for birds of prey using calibrated CT scanning. Estimates for CoM and MoI are provided alongside validation data used to quantify the accuracy of the technique. The advantages of this digital modelling approach are highlighted with novel visualisations of mass distribution in birds of prey. In contrast to the digital modelling method outlined previously, this approach describes mass variation throughout the organism at the level of individual voxels. Birds represent a challenging test case because of their feathered appendages (possibly explaining why inertial properties data for this class are relatively scarce (Mills et al., 2018)). This method should therefore apply equally well to animals with less challenging anatomical features.

## MATERIALS AND METHODS

### Experimental principle

CT scanners measure the attenuation of X-rays through a measurement volume, from multiple orientations, to build a 3D contrast image of the object of interest (Goldman, 2007). The image consists of an array of voxels (3D pixels), each with an intensity dependent on local X-ray attenuation. The attenuation is a function of the atomic composition of the scanned materials and the distance travelled. Modern CT scanners use algorithms such as filtered back projection to provide a measure of the X-ray attenuation in each voxel in Hounsfield units (HU):
(1)

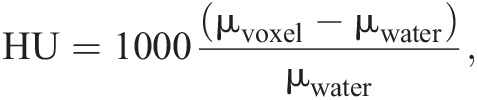
where μ_voxel_ and μ_water_ are the attenuation coefficients of the voxels representing the object of interest and of distilled water, respectively. For biological material, the relationship between the mean absolute density of the tissue in a given voxel and the X-ray attenuation is fairly linear (Mull, 1984). An approximate measure of absolute density, ρ (kg m^−3^) (referred to just as ‘density’ from here on) for biological materials can therefore be obtained from a CT image using:
(2)

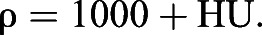


This basic calibration works reasonably well for biological materials with densities close to that of water, and has been used to estimate the mass of human organs (Lescot et al., 2008; Malbouisson et al., 2001). To improve accuracy for densities farther from water, calibrations can be obtained using objects of known density. This approach has yielded accurate mass estimates for different types of wood (Davis and Wells, 1992; Freyburger et al., 2009; Wei et al., 2011). Although calibrated CT has been used successfully for mass estimation in these instances, it should be noted that results can vary depending on the scanner type, model, calibration, settings and scan date (Levi et al., 1982). In particular, the approach assumes that the correlation between X-ray attenuation and object density is linear.

### Experimental animals

Cadavers of naturally deceased barn owls [*Tyto alba* (Scopoli 1769)], peregrine falcons (*Falco peregrinus* Tunstall 1771) and a sparrow hawk [*Accipiter nisus* (Linnaeus 1758)] were used in this study (Table 1). These were obtained from animal rescue centres across the south-west of the United Kingdom and frozen as soon as possible after death in a standard freezer (approximately −18°C) for approximately 1 year. Details of the cause of death and cadaver mass at the time of freezing were provided where possible. As with previous studies using bird cadavers (Kirkpatrick, 1990; Berg and Rayner, 1995), several specimens were emaciated. Only one barn owl cadaver and one peregrine falcon cadaver exhibited a healthy mass based on comparison with records for these species (Robinson, 2005).

**Table 1.**
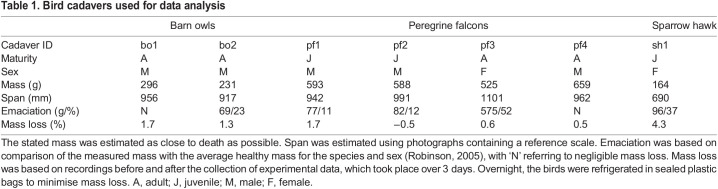
Bird cadavers used for data analysis

### Experimental procedure

#### Cadaver preparation

A trifilar pendulum method was used to validate the calibrated CT estimates of MoI, so it was important to ensure the bird adopted a consistent position and pose during data collection with both techniques. The wings of each cadaver were stretched out and pinned securely (dorsal-side down) to a flat, cross-shaped board. Nylon threads were used to firmly secure the legs, head and primary flight feathers to the board to minimise movement during transportation. A sheet of expanded polystyrene foam (12.5 mm thickness) was placed between the bird and the board to act as a radiolucent cushion to ease segmentation of the CT data. During data collection over several days, the highest mass loss was from the sparrow hawk cadaver (4.3%), with the peregrine falcons and barn owl cadavers losing less than 1.7% mass (Table 1). One cadaver gained 0.5% of its initial mass, which may have been due to moisture absorption by the feathers during handling.

#### CT scanning

**Fig. 1.**
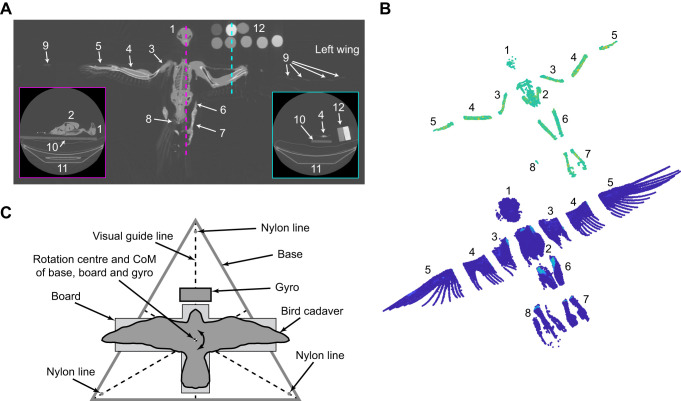
**Computed tomography methods for scanning birds of prey.** (A) Example image slices from the computed tomography (CT) scan of a peregrine falcon showing approximate coronal plane (main) and mediolateral views (insets). (B) Calibrated CT data for the appendage segmented barn owl, bo1, using optimised thresholding to match the calibrated CT mass estimate with the real cadaver (bottom) and a threshold revealing bone (top). The real cadaver was dissected in approximately the same way for validation using appendage mass comparison. (C) Plan view of the trifilar pendulum arrangement. Anatomical labelling: 1, head; 2, keel/pectoralis; 3, humerus; 4, radius and ulna; 5, manus; 6, tibiotarsus; 7, tarsometatarsus and digits; 8, pygostyle and tail rectrices; 9, primary remiges shafts; 10, board; 11, patient support; 12, calibration phantoms.

Each cadaver was CT scanned (LightSpeed RT16, General Electric, Boston, MA, USA) with a voltage and current of 120 kVp and 200 mA, respectively, and a spiral pitch factor of 0.9375. Each image in the stack had a resolution of 512×512 pixels and images were separated by 1.25 mm with pixel widths ranging from 0.68 mm to 0.98 mm depending on the field of view used for each cadaver (these varied depending on the size of the cadaver to maximise use of the measurement volume). Image stacks were extracted in uncompressed 16-bit TIFF format along axes roughly equivalent to the bird's dorsoventral and mediolateral axes (Fig. 1A). The peregrine falcon and barn owl cadavers bo1 and pf4 were also scanned a second time with their wings fully retracted.

#### CT calibration

Linear calibrations (Fig. S1) were generated correlating the voxel grey-values with eight tissue characterisation phantoms (Gammex 467, Sun Nuclear, Melbourne, VIC, Australia) whose densities ranged from 450 to 1820 kg m^−3^. Images from the 25%, 50% and 75% planes, approximately orthogonal to each phantom's longitudinal axis, were used to obtain their mean grey-values by manually drawing an ellipse around each phantom. Regions of air were also sampled in a similar manner. The phantoms were placed next to each bird cadaver, and individual calibrations were generated for each scan. The calibration curves were different between scans/cadavers, possibly due to the different fields of view used to maximise the resolution of each scan.

#### CoM and MoI estimation

To estimate the CoM and MoI tensors, the grey-values from the CT image stack were converted to voxel density and mass using the calibration and voxel volume. The scanned air around the bird was removed using manual thresholding followed by manual segmentation in CloudCompare v2.8.1 (https://www.cloudcompare.org/) to remove unwanted features of the scan (i.e. parts of the CT scanner). The segmentation threshold for the bird was then optimised by minimising the difference between the total cadaver mass estimate from the calibrated scan data (i.e. the sum of the voxel masses) with the known mass of the bird obtained with a mass balance (LE341001P, Sartorius, Göttingen, Germany). This optimised threshold resulted in scan data where the majority of the bird's covert feathers and flight feather shafts remained visible (Fig. 1B) and provided a general indication that the calibration was working as expected (i.e. grey-value thresholding based on total mass yielded the expected visualisation).

The CoM was calculated in Cartesian coordinates using:
(3)

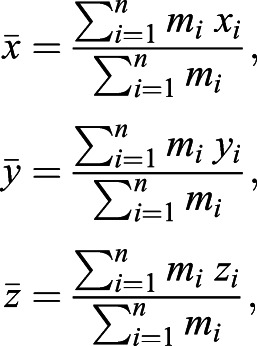
where *m_i_* represents the mass of each of the *n* voxels. For an arbitrary coordinate system, the MoI tensor was calculated using:
(4)

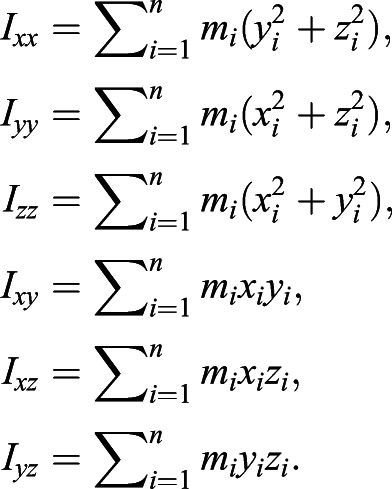
For each scanned bird, the coordinate system origin was translated so that it was coincident with the CoM estimated using the calibrated scan data. The principal axes and components of inertia were then calculated. The principal axes represent a uniquely defined coordinate system with its origin at the CoM with an orientation such that the products of inertia (*I_xy_*, *I_xz_* and *I_yz_*) are zero (Meriam and Kraige, 2012). For a symmetrical object, the principal axes align with the axes of symmetry, and in the case of the scanned birds, this allows for a robust comparison to be made independently of any slight differences in pose or orientation on the board.

#### Appendage mass comparison

In a first approach to validate the accuracy of calibrated CT, comparisons were made between ‘virtual’ and ‘physical’ dissections of the birds’ appendages. All the cadavers, except one peregrine falcon (pf3), were dissected to separate the head and cervical spine, torso, tibiotarsae, tarsometatarsae (including talons) and wings. Each wing was cut at the elbow and wrist joints to separate the humerus, radius/ulna and manus (with associated feathers). The segments were then weighed using a mass balance (LE341001P, Sartorius) to a resolution of 0.1 g. The scan data for these cadavers was manually segmented in the same way as the dissected cadavers (Fig. 1B) and mass was estimated using the CT calibration. A comparison between the mass of the dissected cadaver and segmented scan data was then carried out. From here on, the dissection of the cadaver is referred to as ‘physical dissection’, while the segmentation of the CT scan data is referred to as ‘virtual dissection’.

#### Dorsoventral MoI comparison

In a second approach to validate the accuracy of calibrated CT, the MoI about the birds’ dorsoventral axis (defined as normal to the board) was measured using a trifilar pendulum (referred to as pendulum from here on). Fig. 1C shows the plan view of the pendulum, consisting of an equilateral triangular base suspended by three nylon threads (60 lb fishing line) supporting the board, bird and a gyroscope. The position of the board and gyroscope was determined so that their combined CoM was at the pendulum base CoM. The bird was placed on the board such that its approximate CoM (judged by eye) was as close as possible to this point. A correction for this misalignment between the bird's CoM and the CoM of the remaining pendulum components is described in Eqn 7.

The MoI of the pendulum, *I*_p_, was estimated assuming it behaved as a horizontal, single degree of freedom dynamic system with negligible thread mass (1 mm steel cables did not yield accurate results), rotating about its CoM through small angles such that:
(5)

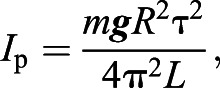
where *m* is the mass of the pendulum, ***g*** is gravitational acceleration, *R* is the distance between the threads and the centre of rotation, τ is the period of oscillation and *L* is the mean length of the threads (Korr and Hyer, 1962). The difference between the pendulum MoI with and without the bird provided an estimate of the bird's MoI about the axis of rotation (without correction for potential misalignment between the bird's CoM and the pendulum CoM). The mass of the bird, board, base and gyro was obtained using an electronic balance (LE341001P, Sartorius) with a resolution of 0.1 g. The angular velocity of the pendulum was sampled at 70 Hz using a smartphone gyroscope (Xperia Z1 Compact, Sony, Tokyo, Japan) and software application to collect the data (Sensor Kinetics Pro, Innoventions Inc., Houston, TX, USA). The curve fit for a damped harmonic oscillator was applied to the last 60 s of approximately 180 s of angular velocity data using Matlab (MATLAB 2017a), based on the following equations (Rao, 1995):
(6)

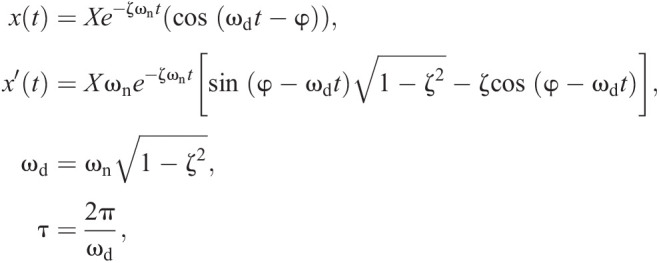
where *x* is angular displacement, *x*′ is angular velocity, *t* is time, ζ is damping coefficient, ω_d_ and ω_n_ are damped and undamped natural frequencies and *X* and ϕ are the amplitude and phase, respectively. The curve fit coefficient corresponding to the damped natural frequency was used to obtain the period of oscillation, τ, and was based on the mean of three repeated measurements in each case.

To compare MoI estimates from the pendulum and calibrated CT data, an axis normal to the board and coincident with the bird's CoM (i.e. dorsoventral) was defined. This dorsoventral axis was aligned to the scanned bird by plane fitting to the scanned board to obtain the correct orientation, followed by translation of the origin to the bird's CoM. The pendulum orientation was matched to this transformation, except for the bird's CoM, which was unknown during its placement on the pendulum. Any misalignment between the bird and pendulum CoM was quantified based on the CT scan data, and was found to range from 8 to 31 mm. The dorsoventral MoI of the bird, accounting for bird CoM misalignment, was then calculated using (du Bois et al., 2009):
(7)

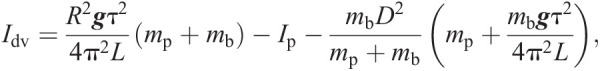
where *I*_dv_ is the dorsoventral MoI of the bird, *m*_b_ and *m*_p_ are the mass of the bird and the pendulum without the bird, respectively, *I*_p_ is the MoI of the pendulum without the bird, τ is the time period of oscillation with the bird and *D* is the CoM offset between the bird and the pendulum without the bird (the remaining variables are as described for Eqn 5).

The pendulum method was validated with machined nylon blocks whose MoI was calculated analytically to very high accuracy (±0.003%, assuming uniform material density). This showed that for objects of equivalent mass and size to a barn owl and peregrine falcon, the MoI using the pendulum was within 3.3% and 0.1% of the analytical estimates, respectively, with the larger error for the barn owl object being due to its smaller mass and dimensions.

## RESULTS AND DISCUSSION

In this section, we present results comparing appendage mass breakdown and dorsoventral MoI between calibrated CT and the two independent methods described previously. Estimates of CoM and the principal components of inertia of the birds used in this study are provided alongside illustrative examples of how calibrated CT can be used to describe mass distribution in birds.

**Fig. 2.**
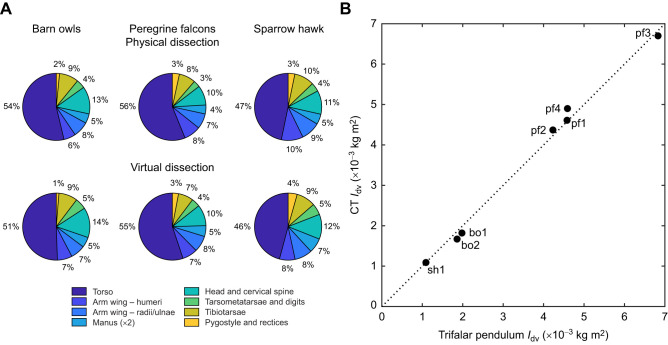
**Validation of the CT approach.** (A) Validation through mass breakdown for the physical versus virtual dissection, averaged for each species (barn owls, *n*=2; peregrine falcons, *n*=3; sparrow hawk, *n*=1). (B) Independent estimates of the dorsoventral moment of inertia (*I*_dv_) from the calibrated CT method and trifilar pendulum (corrected for centre of mass misalignment). Bird ID as in Table 1.

A comparison between the mass breakdown of the physical and virtual cadavers is shown in Fig. 2A, which reveals the effectiveness of calibrated CT for quantifying the overall mass distribution. The relative contributions of the appendages were well matched, with differences typically of 1% or less (versus total cadaver mass) between physical and virtual dissections. Some of the differences may be due to imprecisely matched cut locations between the physical and virtual dissections. In future work, this could be assessed by scanning the physically dissected cadaver.

It should be noted that although the assignment of mass to voxels appears to work relatively effectively, this is not necessarily the case with density. For example, voxels containing several tissue types, including air (such as feather shafts and vanes), yield a density that is a weighted average of all the materials (including air) present within the voxel. This inclusion of air in the volume measurements influences average calculated density measurement and is why other studies focusing on density measurement have removed the feathers (Larramendi et al., 2021).

A comparison is shown in Fig. 2B between the dorsoventral MoI estimated with calibrated CT and the trifilar pendulum for each cadaver. Correcting for the bird's CoM misalignment improved the correlation between the two methods (*R*^2^=0.993, *N*=7). Comparison was also made with wing MoI estimations obtained with strip analysis (Fig. S2; Berg and Rayner, 1995) and showed that the calibrated CT data were consistent with this previously described allometry. Overall, Fig. 2 shows that calibrated CT provides accurate quantification of the global inertial properties in these birds.

We investigated the effect of assuming a uniform density (density=cadaver mass/cadaver volume) for each voxel instead of using a calibration curve to relate grey-value to density. This resulted in up to 5 mm movement of the CoM for the barn owl and peregrine falcon (approximately 5% mean aerodynamic chord; Durston et al., 2019) and an increase of 58% to the dorsoventral MoI. This suggests that volume data alone provide a reasonable estimate for CoM depending on the accuracy required (Macaulay et al., 2017), but lead to more significant errors in MoI from overestimating the mass of the distal appendages.

**Fig. 3.**
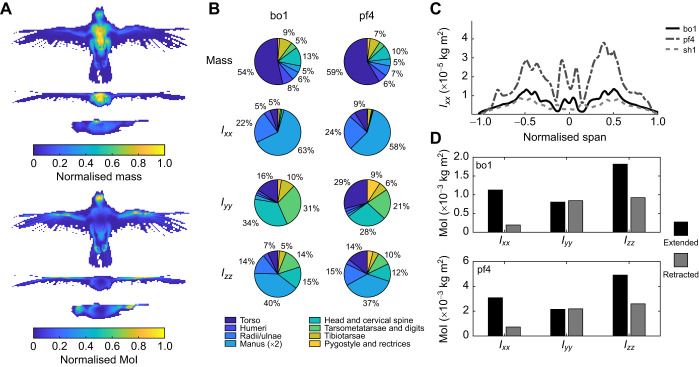
**Visualising avian inertial properties obtained from calibrated CT.** (A) Projected normalised distribution of mass (top) and moment of inertia (MoI bottom) distribution in the peregrine falcon pf4. (B) Relative mass and MoI breakdown by individual cadaver (barn owl bo1, peregrine falcon pf4) from segmentation of the calibrated CT data. *I_xx_*, roll MoI; *I_yy_*, pitch MoI; *I_zz_*, yaw MoI. (C) Distribution of roll MoI versus normalised span for barn owl bo1, peregrine falcon pf4 and sparrow hawk sh1. (D) Principal components of inertia for the barn owl bo1 (top) and peregrine falcon pf4 (bottom) based on CT scan data for wings fully extended and retracted.

Fig. 3A shows dorsoventral, mediolateral and anteroposterior projections of the 3D scan data for the healthy mass peregrine falcon, pf4. The colour maps show mass and MoI sampled and summed on a uniform grid applied to each plane for the top and bottom three views. The data quantify how mass (Fig. 3A, top) and MoI (Fig. 3A, bottom) are distributed for a given viewing direction. In the dorsoventral projection, the mass generally follows the thickness of the cadaver in the view direction. In contrast, local MoI contributions are concentrated more distally in the wing bones, head and feet, highlighting the strong local impact of the distance-squared term in MoI (see Fig. 3C). The anteroposterior view shows mass concentration dorsally, largely due to the dorsal position of the head and legs on the board, and possibly due to the dorsal movement of internal organs (cadavers were dorsal-side down and thawed during scanning).

The relative contributions of the dissected appendages to mass and MoI for cadavers bo1 and pf4 are shown in Fig. 3B and absolute data are given in Tables S1 and S2. The torso contributed 54–59% of the total mass and the wings contributed 18–19% of the total mass, consistent with previous findings (Kirkpatrick, 1990). The tail (pygostyle and rectrices) contributed no more than 4% of the total mass, consisting mostly of feathers. The bird's head was more than half the total mass of the wings and the combined mass of the legs (tibiotarsae, tarsometatarsae and digits) was approximately three-quarters the total mass of the wings.

Fig. 3B also shows significant differences between the mass and MoI contributions of the appendages. Despite contributing only approximately 20% of the total mass, the wing contributed approximately 85% of roll MoI (*I_xx_*) and almost 50% of yaw MoI (*I_zz_*). This effect was particularly evident in the manus (hand wing), which constituted only 5–7% of the total mass, yet contributed 57–63% towards total roll MoI (*I_xx_*) and 32–40% towards yaw MoI (*I_zz_*). Similarly, the head, legs and tail contributed up to 80% of total pitch MoI (*I_yy_*) despite comprising no more than 30% of the total mass. In contrast, the wings contributed only 10% to pitch MoI (*I_yy_*) as a result of their low mass and close proximity to the *y*-axis. About all axes, the contribution of the torso towards all MoI parameters was relatively small (<17%), with the exception of the peregrine falcon's pitch MoI (29% of *I_yy_*). The peregrine falcon's torso contributed slightly more towards total mass (59%) than did the barn owl’s torso (54%). It also had a higher body length to wingspan ratio (0.41) compared with the barn owl (0.34).

Fig. 3C shows the contribution of the wings and body towards roll MoI (*I_xx_*) as a function of normalised wing span (referred to body centreline), similar to plots produced from strip analysis (Berg and Rayner, 1995). Several features match strip analysis findings for a house sparrow (*Passer domesticus*), blackbird (*Turdus merula*) and house swift (*Apus affinis*) (Mohan et al., 1982; Berg and Rayner, 1995), including the ‘double-bell’ feature in the vicinity of the wrist joint and manus. The trough of the double-bell proximally of ±0.5 normalised span locates the intermetacarpal spatium (in the manus) while the peaks locate the wrist joint and the pila cranialis (Baumel et al., 1993). The significant asymmetry in pf4 corresponds to trauma in the right wing, where the locally increased MoI contribution may have been due to excess fluid from inflammatory response.

The principal components of inertia (*I_xx_*, *I_yy_*, *I_zz_*) are shown in Fig. 3D for the healthy weight barn owl (bo1) and peregrine falcon (pf4) cadavers with fully extended and retracted wings. Retracting the wings reduced roll (*I_xx_*) and yaw (*I_zz_*) MoI by approximately 80% and 50%, respectively. In contrast, the pitch MoI and CoM were relatively unchanged between these configurations.

### Conclusions

In this paper, we have described the generation of 3D mass distribution/inertial models for birds of prey using calibrated CT scanning, provided estimates for CoM and MoI including validation with several independent methods and described avian mass distribution using novel visualisations.

As a digital modelling approach, calibrated CT offers clear advantages over physical techniques (i.e. suspension, scales, primitive shapes and strips methods) for estimation of inertial properties in birds. Most of these traditional methods do not easily yield 3D CoM and MoI and require destructive dissection of cadavers that may be difficult to obtain. Calibrated CT is non-destructive and provides 3D inertial properties via datasets that can be manipulated as required (i.e. coordinate transformation, segmentation, thresholding, etc.).

The use of this approach clearly requires access to CT scanners, which are costly to purchase or hire. It is also important to remember that results can vary depending on the scanner, model, calibration, settings and scan date (Levi et al., 1982) and that they rely on a linear relationship between the scanner grey-value output and physical density. Validating data from this method is therefore good practice, not only as a check against these potential pitfalls but also as a check against human error in the experimental procedure. The extent of the validation process need not be as extensive as that provided here; we suggest a mass breakdown comparison with a single cadaver may suffice for future studies. Attention to detail is important; for example, providing a calibration for every scan, including air in the calibration curves, picking scanner settings to maximise contrast and carefully preparing the cadaver.

The animals used in this study, birds of prey, represent a challenging test case mainly because of the delicate nature of flight feathers. This suggests that the method ought to be applicable to a wide range of animals with similar or less challenging anatomy. We hope that this paper leads to widespread use of calibrated CT in biomechanics studies, and that the technique can be used to build a comprehensive database of mass/inertial properties across many taxa for the benefit of the whole research community. In support of this endeavour, it would be worth further testing of this approach, especially with different scanners and species from the scale of insects to larger mammals.

## Supplementary Material

Supplementary information
