# Supplementary material for: Quantifying avian inertial properties using calibrated computed tomography
Source: J Exp Biol. 2022 Jan 4;225(1):jeb242280. doi: 10.1242/jeb.242280 (PMC8778804; doi:10.1242/jeb.242280)
Supplement: Supplementary information [file jexbio-225-242280-s1.pdf]

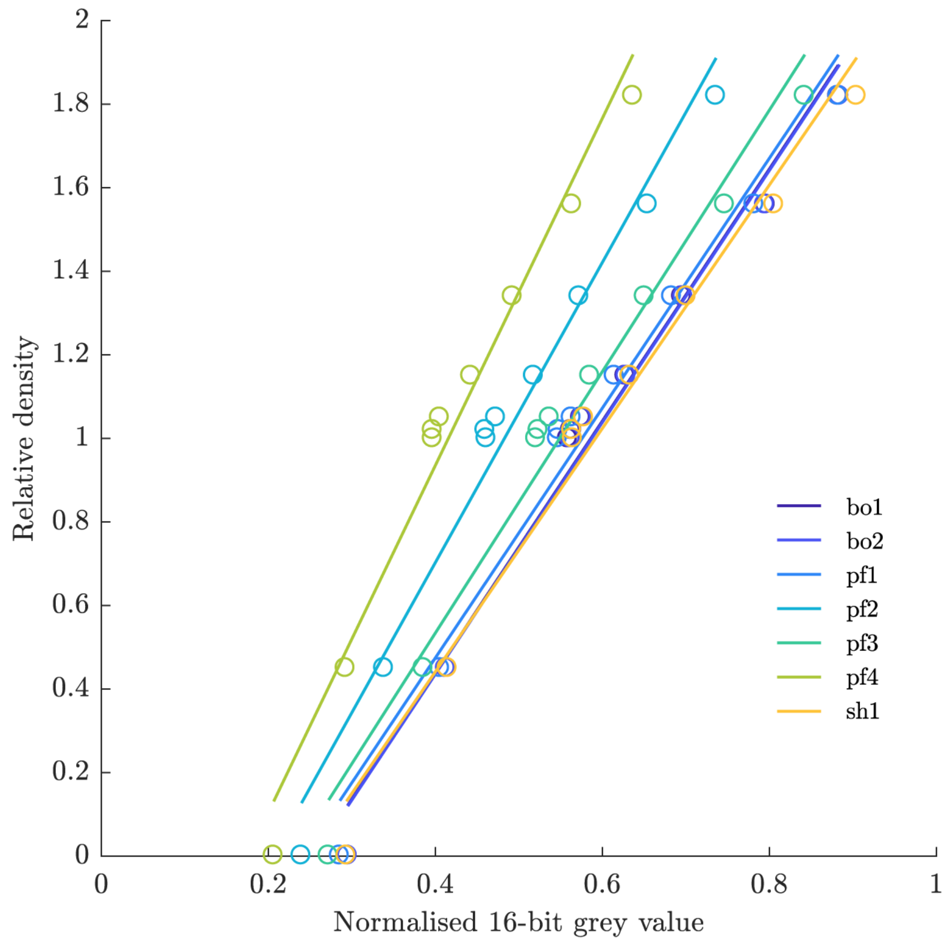

Fig. S1. Linear calibrations for each CT-scanned bird cadaver based on tissue characterisation phantoms (points). The variation in these calibration curves, possibly due to changes in the field of view or the mapping of CT numbers to grey-values, illustrates the importance of obtaining a calibration curve for each individual scan.

Journal of Experimental Biology • Supplementary information

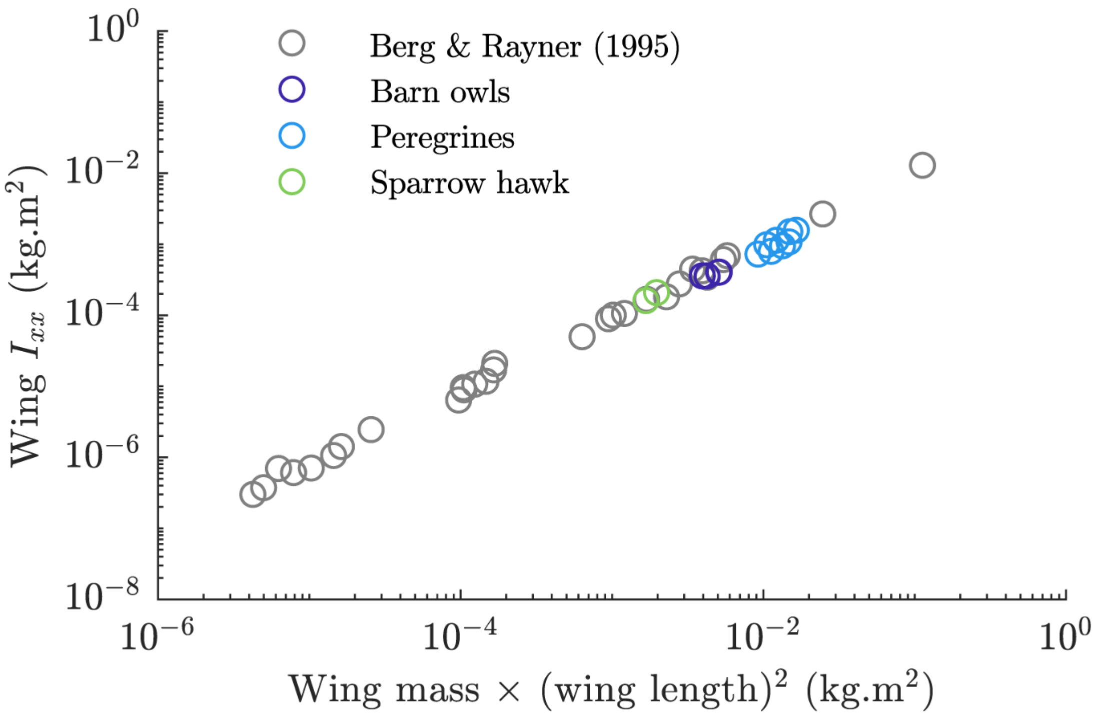

Fig. S2. Comparison between wing MoI measurements using ‘strip analysis’ (Berg and Rayner, 1995) and the present results obtained using calibrated CT. The wing MoI was measured about an axis passing through the humeral head and parallel to the x-axis of the principal axes of inertia.

Journal of Experimental Biology • Supplementary information

Table S1. Body segment properties for healthy weight barn owl specimen (bo1).

Segment	Mass (g)	Volume (cm^3)	Density (kg/m^3)	CoM (mm) x	y	z	I ($kg \cdot m^2$) Ixx	Iyy	Izz
Head & cervical spine	38.8	82.4	471.1	-82.0	13.5	2.9	1.71E-05	2.75E-04	2.81E-04
Torso	156.7	202.1	775.5	4.1	-1.9	-5.1	6.07E-05	1.28E-04	1.29E-04
Humerus (left)	11.5	19.5	590.5	-21.2	-41.4	7.8	2.44E-05	9.29E-06	3.10E-05
Humerus (right)	11.3	18.4	613.7	-16.2	45.7	5.0	2.74E-05	6.84E-06	3.21E-05
Radius & ulna (left)	8.6	17.2	499.6	-13.8	-111.5	16.3	1.14E-04	6.93E-06	1.16E-04
Radius & ulna (right)	8.9	18.4	482.4	1.9	120.5	16.8	1.37E-04	5.36E-06	1.37E-04
Manus (left)	7.1	24.9	285.0	-41.4	-216.6	17.4	3.58E-04	1.76E-05	3.70E-04
Manus (right)	6.5	23.3	280.2	1.8	226.7	17.4	3.55E-04	6.89E-06	3.57E-04
Tibiotarsus	26.1	37.5	695.8	49.8	-4.0	-7.8	1.98E-05	7.71E-05	8.64E-05
Tarsometatarsus & digits	13.4	24.0	559.1	133.2	-13.8	13.6	1.12E-05	2.50E-04	2.56E-04
Pygostyle & tail rectrices	3.9	9.6	401.8	68.3	-6.6	9.0	1.17E-06	2.07E-05	2.08E-05
**Total**	292.9	477.3	613.5	0.0	0.0	0.0	1.13E-03	8.03E-04	1.82E-03

All data based on virtual CT dissection. CoM positions and inertial properties for each segment relative to CoM of whole bird in principal axes of inertia axis system.

Table S2. Body segment properties for healthy weight peregrine falcon specimen (pf4).

Segment	Mass (g)	Volume (cm^3)	Density (kg/m^3)	CoM (mm) x	y	z	I ($kg \cdot m^2$) Ixx	Iyy	Izz
Head & cervical spine	65.9	113.6	579.7	-93.3	-3.2	3.3	1.73E-05	6.07E-04	6.06E-04
Torso	386.7	534.5	723.4	3.7	-2.7	-5.2	2.80E-04	6.24E-04	7.05E-04
Humerus (left)	17.9	29.8	599.4	-27.4	-50.0	14.9	5.24E-05	2.32E-05	6.68E-05
Humerus (right)	21.6	38.5	560.6	-29.9	56.2	8.0	7.63E-05	2.79E-05	9.93E-05
Radius & ulna (left)	22.8	41.5	548.9	-15.3	-115.2	18.4	3.24E-04	1.86E-05	3.26E-04
Radius & ulna (right)	25.2	43.9	574.0	-17.5	125.7	17.3	4.19E-04	2.02E-05	4.23E-04
Manus (left)	14.9	44.3	336.2	-21.8	-227.4	19.7	8.24E-04	1.90E-05	8.29E-04
Manus (right)	17.7	49.5	357.6	-11.5	226.8	19.3	9.69E-04	1.91E-05	9.74E-04
Tibiotarsus	46.6	61.9	752.4	48.0	-1.7	-11.0	8.72E-05	1.36E-04	1.98E-04
Tarsometatarsus & digits	26.4	44.1	598.6	129.6	-5.1	9.1	2.02E-05	4.57E-04	4.72E-04
Pygostyle & tail rectrices	13.4	37.0	361.8	114.2	-2.2	10.0	7.43E-06	1.97E-04	2.00E-04
**Total**	658.8	1038.5	634.4	0.0	0.0	0.0	3.08E-03	2.15E-03	4.90E-03

All data based on virtual CT dissection. CoM positions and inertial properties for each segment relative to CoM of whole bird in principal axes of inertia axis system.
